# Defects in the retina of Niemann-pick type C 1 mutant mice

**DOI:** 10.1186/s12868-014-0126-2

**Published:** 2014-11-29

**Authors:** Xin Yan, Lucy Ma, Marina Hovakimyan, Jan Lukas, Andreas Wree, Marcus Frank, Rudolf Guthoff, Arndt Rolfs, Martin Witt, Jiankai Luo

**Affiliations:** Albrecht-Kossel-Institute for Neuroregeneration, Rostock University Medical Center, Gehlsheimer Strasse 20, D-18147 Rostock, Germany; Institute for Biomedical Engineering, Rostock University Medical Center, F.-Barnewitz Strasse 4, D-18119 Rostock, Germany; Department of Anatomy, Rostock University Medical Center, Gertrudenstrsse 9, D-18055 Rostock, Germany; Electron Microscopy Center, Rostock University Medical Center, Strempelstr. 14, D-18057 Rostock, Germany; Department of Ophthalmology, Rostock University Medical Center, Doberaner Strasse 140, D-18057 Rostock, Germany

**Keywords:** Npc1, Retina, Optic nerve, Neurodegeneration

## Abstract

**Background:**

Niemann-Pick type C1 (NPC1) disease is an inherited lysosomal storage disease caused by mutation of the *Npc1* gene, resulting in a progressive accumulation of unesterified cholesterol and glycolipids in lysosomes of multiple tissues and leading to neurodegeneration and other disease. In Npc1 mutant mice, retinal degeneration including impaired visual function, lipofuscin accumulation in the pigment epithelium and ganglion cells as well as photoreceptor defects has been found. However, the pathologies of other individual cell types of the retina in Npc1 mutant mice are still not fully clear. We hypothesized that horizontal cells, amacrine cells, bipolar cells and glial cells are also affected in the retina of Npc1 mutant mice.

**Results:**

Immunohistochemistry and electron microscopy were used to investigate pathologies of ganglion cells, horizontal cells, amacrine cells, bipolar cells, and optic nerves as well as altered activity of glial cells in Npc1 mutant mice.

Electron microscopy reveals that electron-dense inclusions are generally accumulated in ganglion cells, bipolar cells, Müller cells, and in the optic nerve. Furthermore, abnormal arborisation and ectopic processes of horizontal and amacrine cells as well as defective bipolar cells are observed by immunohistochemistry for specific cellular markers. Furthermore, hyperactivity of glial cells, including astrocytes, microglial cells, and Müller cells, is also revealed.

**Conclusions:**

Our data extend previous findings to show multiple defects in the retina of Npc1 mutant mice, suggesting an important role of Npc1 protein in the normal function of the retina.

**Electronic supplementary material:**

The online version of this article (doi:10.1186/s12868-014-0126-2) contains supplementary material, which is available to authorized users.

## Background

Niemann-Pick type C1 disease (NPC1) is a lysosomal storage disorder induced by mutation of *Npc1* gene and characterized by neuronal degeneration [1–3]. Npc1 is a 13-pass transmembrane protein located in the late-endosome/lysosome and acts as a transporter for sphingolipid/cholesterol trafficking from the late-endosome/lysosome to other organelles and the membrane system [4,5]. The mutation of Npc1 protein causes a progressive accumulation of unesterified cholesterol, phospholipids, glycolipids, and sphingomyelin in lysosomes of multiple tissues, leading to hepatosplenomegaly, tremor, ataxia, dystonia and neurodegeneration.

The Npc1-mutant (Npc1^-/-^) mouse is widely used as an animal model to study NPC1 disease. The Npc1^-/-^ mouse produces progredient neurological symptoms from postnatal day (P) 49 and usually dies at about P80 days of age [6–9]. At the cellular level in the central nervous system (CNS), the Npc1^-/-^ mouse shows an age-related loss of neurons, especially Purkinje cells in the cerebellum and neurons in the cerebral cortex, as well as an increased activation of microglia and astrocytes in different organs and tissues, mimicking phenotypes of NPC1 patients [10–12]. Gliosis and microgliosis have been shown to be especially dominant in the olfactory bulb, which contributes to olfactory deficits [13].

The vertebrate retina is a multi-layer structure comprised of different types of cells. Starting from inside, the innermost layer - the ganglion cell layer (GCL) is formed mainly by cell bodies of retinal ganglion cells (RGCs) and displaced amacrine cells; the inner nuclear layer (INL) is structured by cell bodies of amacrine cells, bipolar cells, horizontal cells, and Müller cells; the outer nuclear layer (ONL) contains cellular bodies of photoreceptors (rods and cones); the inner plexiform layer (IPL) is formed by axons of bipolar cells, dendrites of ganglion cells and processes of amacrine cells, which can be subdivided into five parallel sublaminae (S1 near the INL to S5); the outer plexiform layer (OPL) between the INL and the ONL contains axon terminals of photoreceptors, dendrites of bipolar cells and processes of horizontal cells [14]. All cells in the distinct layers of the retina cooperate with each other to transfer visual information through the optic nerve to the brain.

Ocular involvement has been reported in a wide range of lysosomal storage diseases [15]. For example, in ophthalmological abnormalities, cornea verticillata and lens opacity have been found in Fabry’s disease [16,17] and optical atrophy in Tay-Sachs and Sandhoff diseases [18]. Degenerative changes in the retina have been observed in Gaucher disease and α-mannosidosis [19,20] and almost all parts of the eye have been affected in mucopolysaccharidoses [21].

In the Npc1 animal model, corneal alterations have been found and improved after a combined treatment with miglustat/allopregnanolone [13]. Furthermore, signs of age-related maculopathies, including lipofuscin accumulation in the retinal pigment epithelial layer, photoreceptor degeneration in outer segments, and synaptic layer disruption in the retina, have been reported [22], suggesting an essential role of Npc1 protein in normal retinal function. In the present study, we further investigated individual cellular pathologies of the retina in the Npc1^-/-^ mouse. Our results showed that electron-dense inclusions are accumulated in different types of cells, and ectopic processes of horizontal and amacrine cells form aberrant arborisation. Furthermore, hyperactivity of glial cells is also revealed. The various patterns of alterations presented in our data suggest multiple cellular defects in the Npc1^-/-^ retina.

## Methods

### Animals

Npc1^-/-^ and control wild type (Npc1^+/+^) mice were bred using heterozygous pairs (BALB/cNctr-*Npc1*^m1N^/J), purchased from the Jackson Laboratory (Bar Harbor, ME, USA). Mice were killed by cardiac perfusion with phosphate-buffered saline (PBS), followed by 4% paraformaldehyde (PFA) in 0.1 M PBS after deep anesthesia with pentobarbital. After enucleation, eyes were fixed in 4% PFA overnight, and stored at -80°C until further processing. At least 3 samples from different mice for each genotype were analyzed at P65. All animal experiments were approved by the local ethics committee (Landesamt für Landwirtschaft, Lebensmittelsicherheit und Fischerei Mecklenburg-Vorpommern; Ref.# 7221.3-1.1-030/12) and performed according to the rules and regulations of the local ethical committee and the Care and Use of the Laboratory Animals.

### Genotype analysis

For identification of mice genotype, genomic DNA extracted from about 1 mm long tail of mice with phenol-chloroform was used as a template for polymerase chain reaction (PCR) according to the protocol described previously [2] using the primers suggested by the Jackson Laboratory.

### Immunohistochemistry

Fluorescent immunohistochemistry was performed on cryostat sections using the method as described previously [23]. Primary mouse monoclonal, rat monoclonal or rabbit polyclonal antibodies raised against calbindin (Swant, Marly, Switzerland; 1:1000), CD68 (Serotec, Raleigh, NC; 1:500), glial fibrillary acidic protein (GFAP; DAKO, Carpinteria, CA; 1:300), glutamine synthetase (Abcam, Cambridge, MA; 1:1000), alpha subunit of G protein (Goα; Millipore, Billerica, MA; 1:1000), hexaribonucleotide binding protein-3 (NeuN; Millipore; 1:100), Neurofilament (SMI32; Covance, Munich, Germany; 1:1000), Npc1 (Abcam; 1:300), alpha isoform of protein kinase C (PKCα; Sigma; 1:1000), tyrosine hydroxylase (Millipore; 1:1000), and a microglia marker (F4/80; 1:200) were used. All primary antibodies are specific to determine their target proteins, which were used as specific markers to identify different types or structures of cells. Alexa 488-labeled (Molecular Probes, Eugene, USA) or Cy3-labeled (Dianova, Hamburg, Germany) secondary antibodies against mouse, rat or rabbit IgG were used. Fluorescence in sections was detected by a confocal microscope (Zeiss LSM780; Jena, Germany). Digital images were adjusted in contrast and brightness with the Photoshop software (Adobe Systems, San Jose, USA).

### Cell apoptosis analysed by TUNEL assay

Terminal deoxynucleotidyl transferase dUTP nick end labeling (TUNEL) assay was performed on cryostat sections using the In Situ Cell Death Detection Kit/TMR red (Roche, Penzberg, Germany) according to the manufacturer’s instructions.

### Transmission electron microscopy

For electron microscopy, the eyes were removed and fixed by immersion in 0.1 M cacodylate buffer containing 2.5% glutaraldehyde for at least 24 hours at 4°C. Subsequently, the retina was dissected into quadrants, osmicated, washed, block contrasted with 2% aqueous uranyl acetate, dehydrated through a graded series of ethanol, and embedded in Epon 812 (Plano GmbH, Marburg, Germany). Ultrathin sections (about 70 nm) were mounted on pioloform-coated slot copper grids and contrasted with uranyl acetate (4 minutes) and lead citrate (2 minutes). The specimens were examined with a Zeiss EM 902 transmission electron microscope (Zeiss, Oberkochen, Germany) at 80 kV. Photographs were taken using a CCD camera (Proscan, Lagerlechfeld, Germany) and adjusted using Photoshop CS2 software (Adobe Systems).

## Results

### Npc1 expression and cell apoptosis in retina

**Figure 1:**
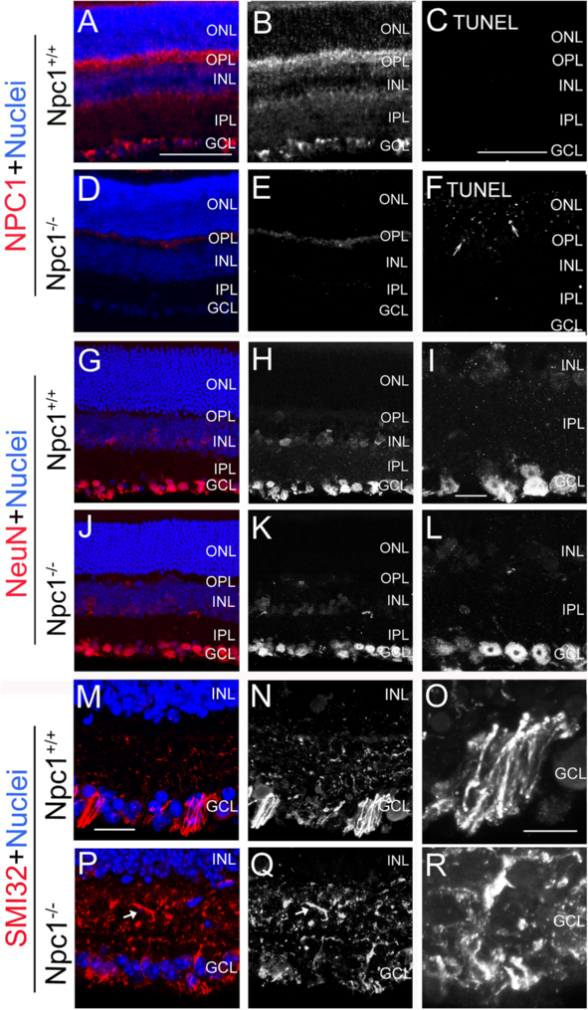
**Abnormal expression of Npc1 protein, the number of apoptotic cells and defective retinal ganglion cells in the Npc1** ^**-/-**^ **retina.** Retinal sections from postnatal day (P) 65 Npc1^+/+^ and Npc1^-/-^ mice were immunostained with antibodies against Npc1 **(A, B, D, E)**, NeuN **(G-L)**, SMI32 **(M-R)**, and terminal deoxynucleotidyl transferase dUTP nick end labelling (TUNEL) assay (white in **C, F**). Nuclei were stained with DAPI (blue). **I, L, O, R** show the magnification of the inner part of retina from **H, K, N, Q**, respectively. Abbreviations: ONL, outer nuclear layer; OPL, outer plexiform layer; INL, inner nuclear layer; IPL, inner plexiform layer; GCL, ganglion cell layer. Scale bar: 5 μm in **O** for **O, R**; 10 μm in **I** for **I, L** and in **M** for **M, N, P, Q**; 50 μm in **A** for **A, B, D, E, G, H, J, K** and in **C** for **C, F**.

We firstly analyzed Npc1 protein expression by immunohistochemistry in the retina of P65 mice. Endogenous Npc1 protein in the Npc1^+/+^ mouse was expressed strongly in the OPL, which consists of the synapses among bipolar cells, photoreceptor cells, and horizontal cells, and by ganglion cells in the GCL (Figure 1A,B). Furthermore, signals were also detected moderately in the IPL, which consists of cellular processes of bipolar cells, ganglion cells and amacrine cells, and weakly in the outer and inner part of the INL, which contains horizontal cells and amacrine cells, respectively, as well as moderately in the outer part of the IPL (Figure 1A,B). In contrast, in the Npc1^-/-^ mouse only weak Npc1 background was found in the OPL (Figure 1D,E). Taken together, Npc1 protein was endogenously expressed by ganglion cells, horizontal cells, amacrine cells, bipolar cells and their processes.

We further evaluated cell apoptosis in the retina of P65 mice by TUNEL assay. Only a few apoptotic cells were detected in the Npc1^+/+^ mouse (Figure 1C), but the number of apoptotic cells found in the Npc1^-/-^ mouse was significantly higher, especially in the ONL and OPL (e.g., arrows in Figure 1F). However, the number of apoptotic cells found in the GCL of the Npc1^-/-^ mouse was very low, showing a similar pattern like the Npc1^+/+^ mouse (compare Figure 1C and Figure 1F).

### Defects in retinal ganglion cells and electron microscopy findings

**Figure 2:**
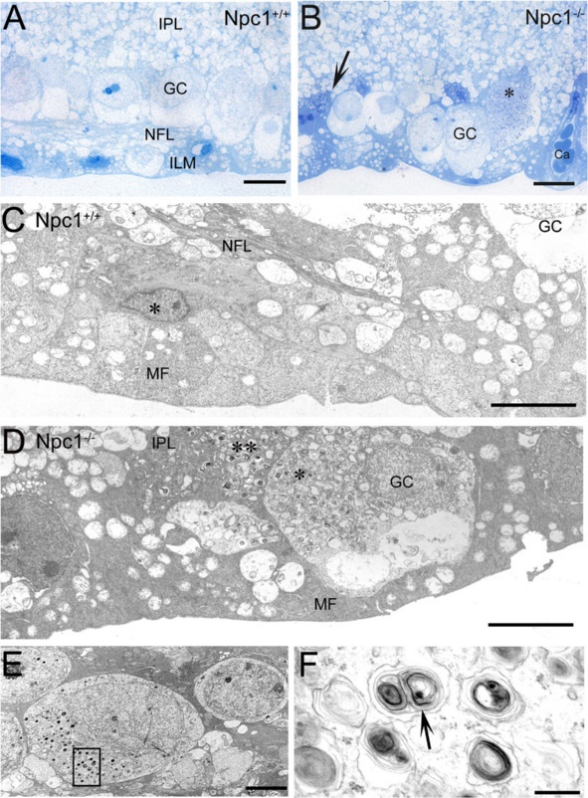
**Semithin sections and electron micrographs of the P65 Npc1** ^**+/+**^ **and Npc1** ^**-/-**^ **retina. A**, **B**: Semithin sections; A shows regular retinal formation with internal plexiform layer (IPL), ganglion cell layer (GC), nerve fiber layer (NFL) and inner limiting (glia) membrane (ILM); B: In the Npc1^-/-^ retina, a large ganglion cell (asterisk) and smaller dark stained extensions of a Müller cell (arrow) contain deposits; C-F: Transmission electron microscopy of the inner layers of Npc1^+/+^ retina **(C)** and Npc1^-/-^ retina **(D)**. The nucleus marked with an asterisk in C is either an astrocyte or an “untypical Müller cell” located on the periphery of the fovea [46]. The organization of Müller cell endfeet seems disturbed in Npc1^-/-^ retina **(D)**. Dark cytoplasmic extensions of Müller cells contain autophagosomes (** in **D**) and the same is visible in somata of a ganglion cell (GC; * in **D**). E shows a representative ganglion cell containing many large myelin-like deposits in phagolysosomes. The rectangle refers to a magnification in **F**. The arrow points to myelin deposits within a membrane-bound compartment. Abbreviations: Ca, capillary; MF, Müller cell endfeet, NFL, nerve fiber layer. Scale bars: 10 μm in **A**, **B**; 5 μm in **C, D**; 2.5 μm in **E**; 250 nm in **F**.

Neurodegeneration including axonal and synaptic abnormalities in the brain of Npc1^-/-^ mice has been reported [24]. To examine effects of Npc1 mutation on ganglion cells in the mouse retina, immunohistochemistry was performed using antibodies against NeuN for ganglion cell soma [25,26] and neurofilament (SMI32) for α-ganglion cell dendrites [27]. The results showed that the distribution of NeuN-positive neurons in both Npc1^+/+^ and Npc1^-/-^ mice was similar, i.e., a large majority of NeuN-positive cells were located in the GCL and a few in the inner part of INL (Figure 1G-L). However, semithin sections and transmission electron microscopy revealed that many ganglion cells in Npc1^-/-^ mice contained electron-dense myelin-like inclusions (Figure 2B, D-F) when compared to the wild type mouse (Figure 2A,C). Furthermore, the expression patterns of neurofilament-positive dendrites by SMI32 staining [27] were different between the groups. In Npc1^+/+^ mice, neurofilament-positive processes were strongly expressed in the GCL and moderately in the inner part of the IPL (Figure 1M-O), but in Npc1^-/-^ mice, expression was strongly in the whole IPL (Figure 1P,Q), suggesting a defective arborisation of lamina-specific dendrites of ganglion cells in the Npc1^-/-^ retina.

**Figure 3:**
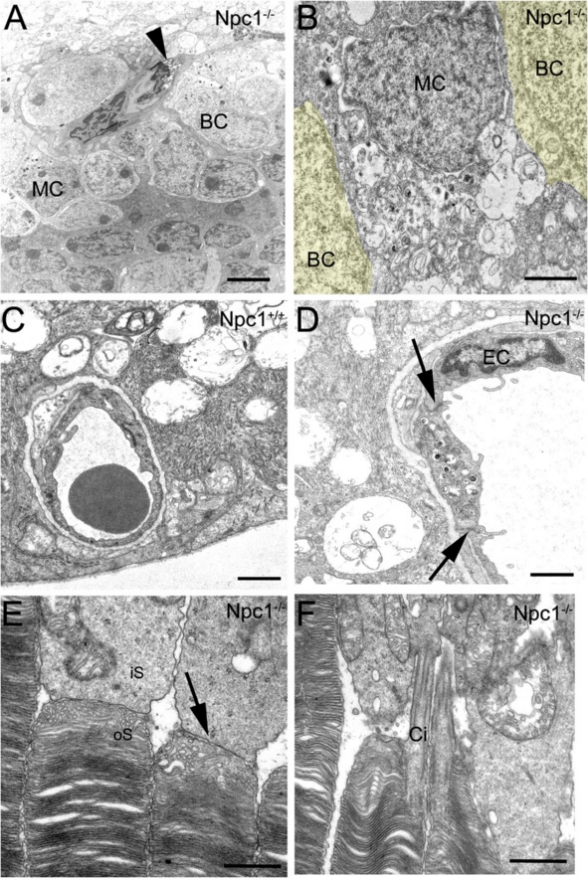
**Electron micrographs of Npc1** ^**-/-**^ **retinae. A**, **B**: In the inner nuclear layer (INL), bipolar cells (BC) and darkly stained Müller cell (MC) as well as endothelial cell (arrowhead in **A**) contain electron- dense deposits. Bipolar cells in **B** are marked yellow. **C, D**: Electron- dense deposits are also found in the endothelial cell (EC) of the Npc1^-/-^ retina **(D)**, but not in wild type animals **(C)**. Tight junctions in the capillary between endothelial cells appear to be intact (arrows in **D**). **E**, **F**: In the outer nuclear layer (ONL) of the Npc1-/- retina, the interface area (arrow) between inner (iS) and outer (oS) segments **(E)** and the cilia (Ci) linking outer and inner segments **(F)** of photoreceptor cells appear normal. In contrast to earlier observations detecting with immunohistochemistry [22], we did not encounter disorganized membrane stacks of oS. Scale bars: 2.5 μm in A; 1 μm in **B-D**; 500 nm in **E**, **F**.

At the electron microscopical level, somata and processes of Müller cells (MC; arrows in Figure 3A,B) and endothelial cells of capillaries (Figure 3D) in Npc1^-/-^ mice were affected by accumulation of electron-dense inclusions, but not in the wild type mouse (Figure 3C). However, despite the numerous autophagosome inclusions in endothelial cells, the tight junctions between endothelial cells to define the blood-retina barrier seemed morphologically unaltered (arrows in Figure 3D). Furthermore, photoreceptor cells located in the ONL did not contain myelin-like materials in Npc1^-/-^ tissues (Figure 3E) and the interface between inner and outer segments as well as the stabilizing ciliar apparatus remained intact (Figure 3E,F), although earlier observation [22] shows that we did not encounter disorganized membrane stacks of oS.

### Defects in horizontal and amacrine cells

**Figure 4:**
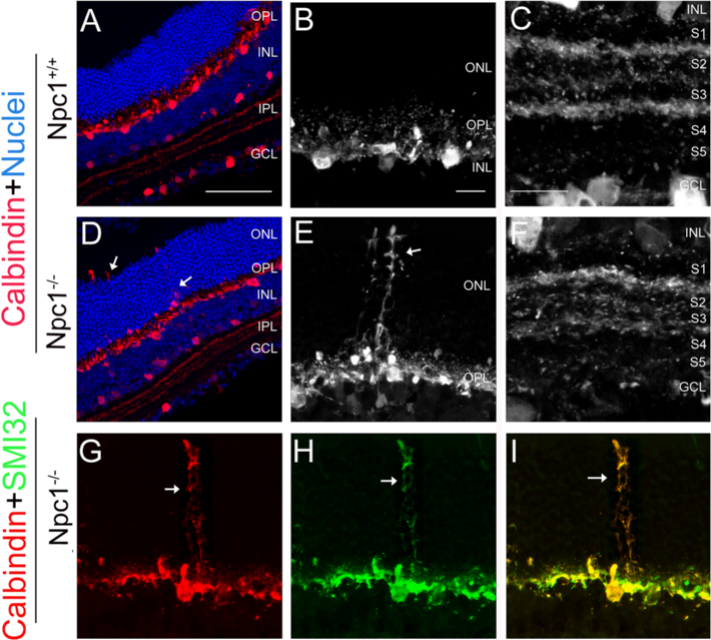
**Defective in horizontal cells in the Npc1** ^**-/-**^ **retina. A-F**: Retinal sections from P65 Npc1^+/+^ **(A-C)** and Npc1^-/-^ **(D-F)** mice were immunostained with an antibody against calbindin (red). Nuclei were stained with DAPI (blue). **B, E** show magnification of the ONL from **A, D,** and **C, F** of the inner part of the retina from **A, D,** respectively. **G-I**: double immunostaining with antibodies against calbindin (red in **G**) and SMI32 (green in **H**) revealed ectopically extended axonal terminal-like structures of the Npc1^-/-^ horizontal cells (yellow in **I**). Abbreviations: ONL, outer nuclear layer; OPL, outer plexiform layer; INL, inner nuclear layer; IPL, inner plexiform layer; GCL, ganglion cell layer; S1-S5, five parallel sublaminae of INL. Scale bar: 10 μm in B for **B, E, G-I** and in **C** for **C** and **F**; 50 μm in **A** for **A** and **D**.

Horizontal cells, subtypes of amacrine cells as well as some ganglion cells in the retina are immuno-positive for calbindin [22,28]. Generally, the distribution of calbindin-positive horizontal and amacrine cells was similar in the outer layer or the inner layer of the INL in both groups (Figure 4A-F). Amacrine and ganglion cells seemed to extend their projections to the borders of S1 to S3 in the IPL (Figure 4C,F) in both Npc1^+/+^ (Figure 4A-C) and Npc1^-/-^ (Figure 4D-I) retinae. However, in the Npc1^-/-^ retina, some neurites of horizontal cells extended ectopically upwards through the ONL and reached the outermost edge of the ONL (arrows in Figure 4D,E). Remarkably, these dorsal-vertical extending neurites co-expressed neurofilament and exhibited axon terminal-like structures (arrows in Figure 4G-H), suggesting that they belonged to axons of horizontal cells as described previously [29].

**Figure 5:**
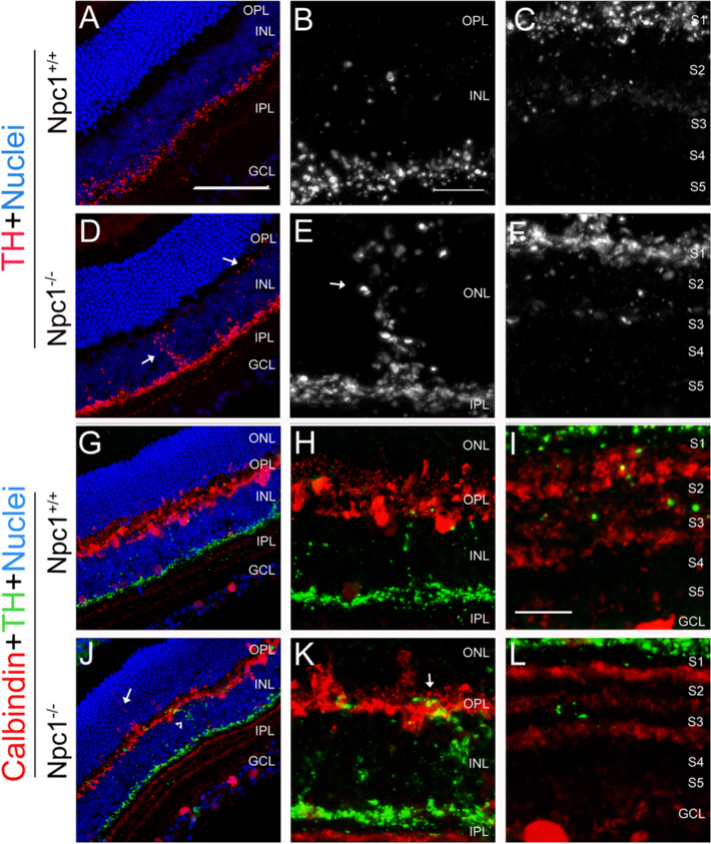
**Defects in amacrine cells in the Npc1** ^**-/-**^ **retina. A-F**: Retinal sections from P65 Npc1^+/+^ **(A-C)** and Npc1^-/-^ **(D-F)** mice were immunostained with an antibody against tyrosine hydroxylase (TH, red). Nuclei were stained with DAPI (blue). **B, E** show magnification of the INL of the retina from **A, D,** and **C, F** of the IPL from **A, D**, respectively. **G-L**: Double immunostaining with antibodies against calbindin (red in **G-L**) and TH (green in **G-L**) revealed ectopically extended neurites of the Npc1^-/-^ amacrine cells (arrows in **J, K**; yellow in **K**). Abbreviations: ONL, outer nuclear layer; OPL, outer plexiform layer; INL, inner nuclear layer; IPL, inner plexiform layer; GCL, ganglion cell layer; S1-S5, five parallel sublaminae of INL. Scale bars: 10 μm in **B** for **B, C, E, F, H, K**, and in **I** for **I, L**; 50 μm in **A** for **A, D, G, J**.

Tyrosine hydroxylase (TH) is a marker for dopaminergic amacrine cells in the retina [28]. In the Npc1^+/+^ retina, TH-positive processes of amacrine cells were found predominantly in the S1 sublamina of the IPL (Figure 5A-C). However, in the Npc1^-/-^ retina, some stronger ectopic TH-positive processes were found to extend to the INL and OPL (arrows in Figure 5D,E). Furthermore, double immunostaining showed that ectopic TH-positive processes of amacrine cells extending into the OPL co-expressed calbindin (yellow, arrow in Figure 5K), but not in the Npc1^+/+^ retina (Figure 5G-I), suggesting that ectopic processes formed synapses with calbindin-positive horizontal cells in the Npc1^-/-^ retina accompanied by a defective lamina-specific arborisation of amacrine cells in the OPL.

### Defects in bipolar cells

**Figure 6:**
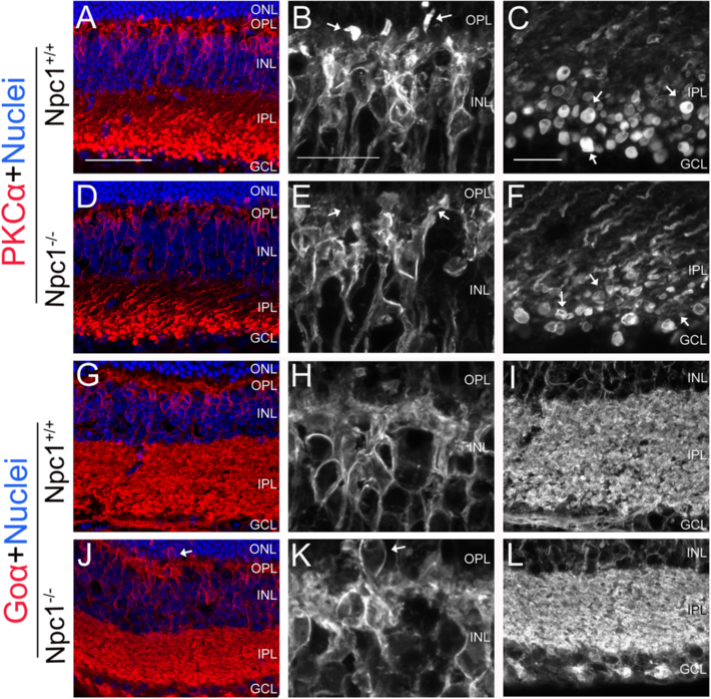
**Defected bipolar cells in the Npc1** ^**-/-**^ **retina.** Retinal sections from P65 Npc1^+/+^ and Npc1^-/-^ mice were immunostained with antibodies against PKCα **(A-F)** and Goα **(G-L)**. Nuclei were stained with DAPI (blue). **B, E, H, K** show magnification of the INL of retina from **A, D, G, J,** respectively. **C, F, I, L** show magnification of the INL from **A, D, G, J,** respectively. Abbreviations: ONL, outer nuclear layer; OPL, outer plexiform layer; INL, inner nuclear layer; IPL, inner plexiform layer; GCL, ganglion cell layer. Scale bar: 10 μm in **B** for **B, E, H, K** and in **C** for **C, F, I, L**; 40 μm in **A** for **A, D, G, J**.

Rod bipolar cells can be labeled by a specific antibody against PKCα [30]. In the Npc1^+/+^ retina, rod bipolar cells, located in the outer part of the INL, had large dendritic arbors to make contact with rod photoreceptors in the OPL (arrows in Figure 6B) and formed axonal endings with large terminal end-bulbs in the IPL (arrows in Figure 6C). In the Npc1^-/-^ retina, however, the dendritic branches were less profuse and some cells were almost devoid of dendrites (arrows in Figure 6E). Furthermore, axonal terminal arbors of rod bipolar cells were reduced in size (arrows in Figure 6F). As a comparison, cone bipolar cells, which receive their input from cone photoreceptors via dendritic arbors, were immunostained with Goα antibody [31]. Our results (Figure 6G-L) indicated that cell bodies of cone bipolar cells in the Npc1^-/-^ retina were ectopically distributed in the ONL (arrow in Figure 6K). Although the intensity of the dendritic immunoreactivity in both types of retina was similar in the IPL (Figure 6G-L), the dendritic arborization of Goα-positive cells was thinner in the Npc1^-/-^ retina (Figure 6J,L).

### Activated glial cells

**Figure 7:**
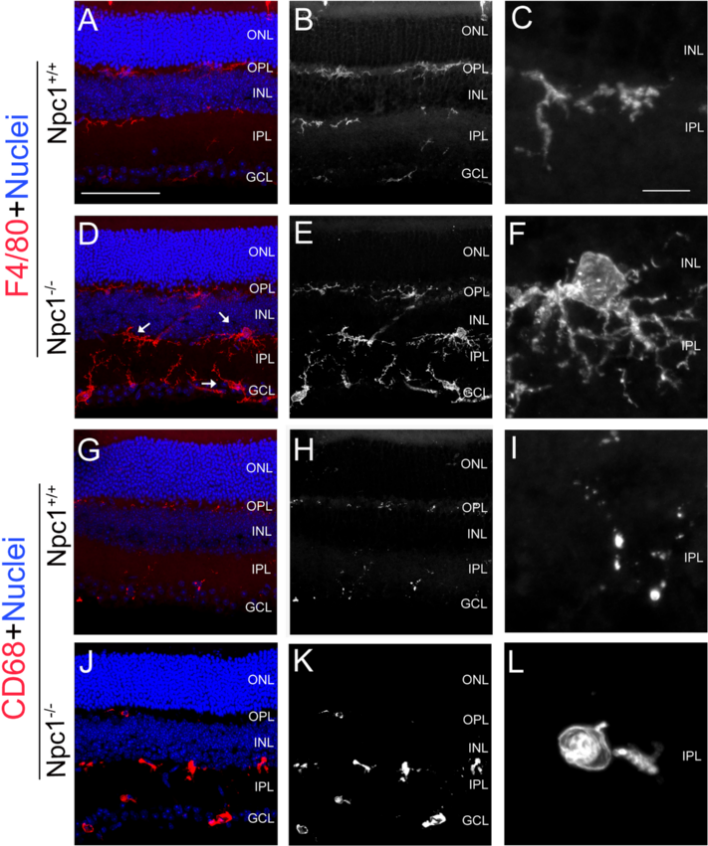
**Activated microglial cells in the Npc1** ^**-/-**^ **retina.** Retinal sections from P65 Npc1^+/+^ and Npc1^-/-^ mice were immunostained with antibodies against F4/80 **(A-F)** and CD68 **(G-L)**. Nuclei were stained with DAPI (blue). **C, F, I, L** show magnification of immuno-positive areas from **A, D, G, J,** respectively. Abbreviations: ONL, outer nuclear layer; OPL, outer plexiform layer; INL, inner nuclear layer; IPL, inner plexiform layer; GCL, ganglion cell layer. Scale bar: 10 μm in **C** for **C, F, I, L**; 50 μm in **A** for others.

In the Npc1^+/+^ retina at P65, a few microglial cells were labeled with antibodies raised against F4/80 (Figure 7A-C) and CD68 (Figure 7G-I), which are markers for microglial cells [32]. In contrast, in the Npc1^-/-^ retina, both number and size of F4/80- or CD68-positive microglial cells were increased in the IPL and the GCL from P9 onwards (see Additional file 1: Figure S1) and strong signals were retained at P65 (Figure 7D-F, J-L). These microglial cells had a larger cell body with strong processes compared to the wild-type microglia (Figure 7A-C, G-L), suggesting an increase of glial cell activity.

**Figure 8:**
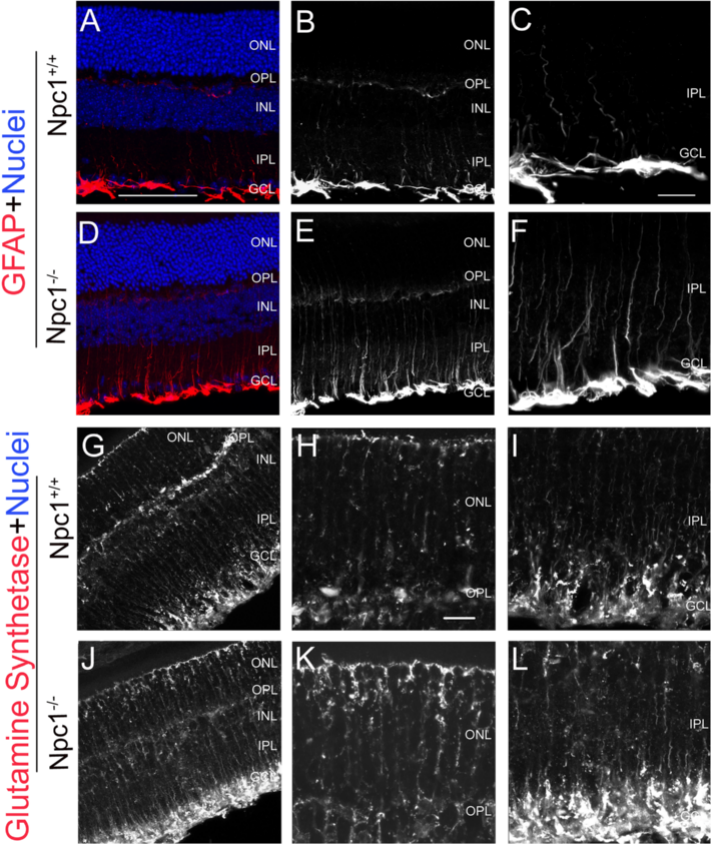
**Activated astrocytes and Müller cells in the Npc1** ^**-/-**^ **retina.** Retinal sections from P65 Npc1^+/+^ and Npc1^-/-^ mice were immunostained with antibodies against GFAP **(A-F)** and glutamine synthetase **(G-L)**. Nuclei were stained with DAPI (blue). **C, F** show magnification of immuno-positive areas from **A, D,** respectively. **H, K** show magnification of the ONL, and **I, L** of the IPL and GCL from **G, J,** respectively. Abbreviations: ONL, outer nuclear layer; OPL, outer plexiform layer; INL, inner nuclear layer; IPL, inner plexiform layer; GCL, ganglion cell layer. Scale bar: 10 μm in **C** for **C, F** and in **H** for **H, I, K, L**; 50 μm in **A** for **A, B, D, E, G, J**.

For astrocytes, GFAP-positive cells were limited to the GCL but their processes appeared weakly in the IPL in the Npc1^+/+^ retina at P65 (Figure 8A-C). However, in the Npc1^-/-^ retina, GFAP signals were increased in the GCL from P9 onwards, and strong signals were found in the GCL and OPL from P30 onwards (see Additional file 1: Figure S1). At P65, GFAP expression was strong in both cellular bodies of astrocytes and their inner processes (Figure 8D-F).

Glutamine synthetase (GS) is a protein that catalyzes ammonia and glutamate into glutamine and mainly exists in Müller cells of the retina [33]. Here, in the Npc1^+/+^ retina, GS immunoreactivity was detected mainly in the cell bodies of Müller cells (Figure 8G,H), but in the Npc1^-/-^ retina, expression was predominantly found in the inner processes of Müller cells, especially in the inner end-feet (Figure 8J-L), showing a shift of expression from soma to inner end-feet.

### Defects in the optic nerve

**Figure 9:**
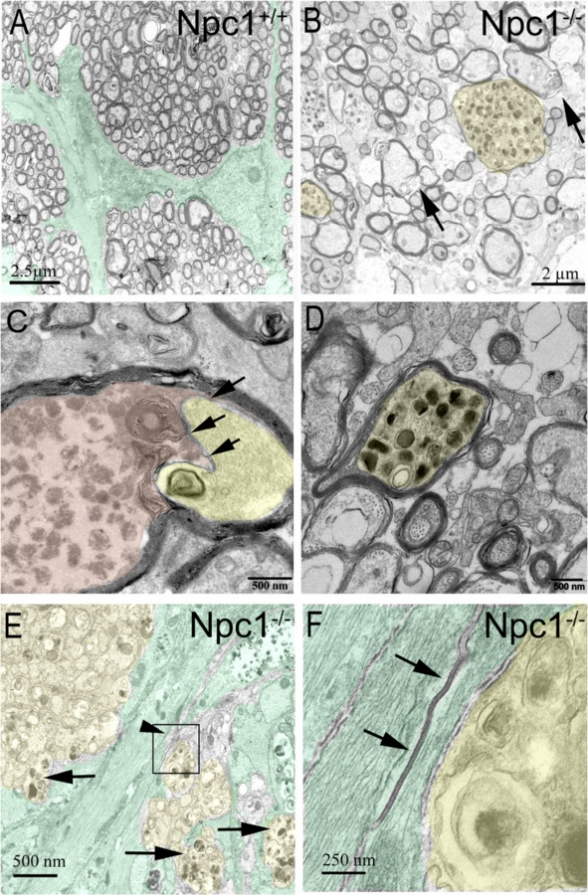
**Transmission electron microscopy of the optic nerve in Npc1** ^**+/+**^ **(A) and Npc1** ^**-/-**^ **(B-F) animals. A**: Regular bundle arrangement in the Npc1^+/+^ optic nerve. Two astrocytes with primary processes enwrapping numerous myelinated optic axons are labelled green. **B**: Individual fiber bundles of optic nerve filled with inclusion material in the Npc1^-/-^ mouse (yellow). Arrows point at two examples of disturbed myelination pattern. **C**: An axon (yellow-colored) with regular arrangement of neurofilaments and one probably irregular myelin-like inclusion body is clearly demarcated by its cell membrane and even indented by the autophagic material of the adjacent oligodendrocyte (weak red). In contrast to Figure 9D, the immediate surrounding cell is loaded with autophagic material and enwraps the axon completely. **D**: The axon is filled with inclusion material, whereas the myelin sheath remained intact (yellow). **E**: Large glia bundles (green), some of which contain inclusions in the Npc1^-/-^ mouse; yellow color shows the nerve fibers bundles with myelin-like material (arrows). Elongated gap junctions appear morphologically intact (arrowhead). **F**: Magnification of the area outlined by a rectangle in **E**; Two adjacent astrocytes are connected with apparently intact gap junctions (arrows). Scale bar: 500 nm in **C**.

The optic nerve comprises bundles of differently myelinated axons of retinal ganglion cells. Myelination starts approximately 0.8-1 mm after leaving the globe [34]. In Npc1^+/+^ mice, the optic nerve was surrounded by a pial sheath consisting of collagen fibers (Figure 9A), and the individual nerve fiber bundles were enveloped by processes of astrocytes (green in Figure 9A). However, in Npc1^-/-^ mutants, we observed defects in myelination (arrows in Figure 9B) as well as obvious and numerous autophagosome inclusions within partly extremely dilated axons (yellow in Figure 9B-D), although some autophagosomes are seen in axons with normal myelin (yellow in Figure 9D). Oligodendrocytes (purple in Figure 9C) and astrocytes (green in Figure 9E) were also affected, but astrocytes maintained connectivity to each other by morphologically intact gap junctions (arrows in Figure 9F).

## Discussion

In the Npc1^-/-^ retina, some disorders, e.g., reduced photo-transduction and photoreceptor synaptic transmission, degeneration of photoreceptor cells and increased autophagy in the GCL, have been described previously [22,35]. In the present study, our results extend previous findings [22] to demonstrate the extensive aberrations, such as electron-dense inclusions accumulated in ganglion cells, bipolar cells, and Müller cells (Figures 2 and 3), abnormal arborisation and misaligned dendrites of horizontal and amacrine cells (Figures 4 and 5), defective bipolar cells (Figure 6), and hyperactive glial cells (Figures 7 and 8; also see Additional file 1: Figure S1).

The ganglion cells in the retina are important for transfer of light information from the retina to the brain. In this study, we show that the number and distribution of ganglion cell bodies in the GCL are similar in both Npc1^+/+^ and Npc1^-/-^ retinae, however, the dendritic arbor distribution of ganglion cells in the IPL of Npc1^-/-^ retina is dispersed from the position of ON-α-ganglion to OFF-α-ganglion (Figure 1). Furthermore, alterations in myelination and numerous autophagosome inclusions in dilated axons of the optic nerve (Figure 9) are also found. These defects may contribute to reduced photo-transduction in Npc1^-/-^ mice.

Horizontal cells and amacrine cells are interneurons that can synapse with photoreceptors, bipolar cells and ganglion cells in the retina, respectively [36]. The aberrant neurite outgrowth of retinal cells with ectopic contact formation is a common feature in mice with defective synaptic transmission from photoreceptor to bipolar cells [37]. Under the degeneration condition, retinal cells can extend their axons and ectopically establish synapses with photoreceptors residing far from these cells [38,39]. Here, we found that the horizontal cell processes, which normally synapse with photoreceptor axon terminals in the OPL, ectopically target to the ONL. Furthermore, the amacrine cell processes also ectopically extend to the OPL and form an aberrant connection with horizontal cells in the Npc1^-/-^ retina, suggesting that the stratification of retinal neurites is altered (Figures 4 and 5). Different guidance molecules, such as semaphorins and plexins, control such neurite stratification in the retina [28,29]. For example, semaphorin3A, expressing throughout the retina and optic nerve during embryonic and postnatal stages [40], is involved in the axon elongation of retinal ganglion cells [41]. Genetic deletion of semaphorin3A results in defects of neurite arborisation, lamination and synapse formation [28,29]. Interestingly, lipid rafts can mediate guidance actions of semaphorin3A and affect neurite outgrowth in the CNS [42]. NPC1 mutation causes alterations of lipid raft, inducing disruptions in the processing and functioning of raft-associated proteins on the plasma membrane of cells [43,44]. Therefore, our data suggest that the neurite stratification in retina relies on, at least in part, Npc1 function. Whether Npc1 is involved in the modulation of axonal guidance cues, e.g., semaphorins, during neurite stratification of the mouse retina should be further investigated.

Activities of glial cells, including astrocytes, microglia, and Müller cells, are up-regulated in the Npc1^-/-^ retina (Figures 7 and 8, also see Additional file 1: Figure S1). Displaced Müller cells have been observed in experimental hypercholesterinemia in the ganglion cell layer of rabbits [45], but occur also physiologically near the fovea centralis (Figure 3C) [46]. Gliosis is a common feature for ongoing neurodegeneration and plays an important role in pathologies of NPC1 disease [12,24]. Therefore, our data suggest that hyperactivity of glial cells contributes to the development of neurodegeneration processes in the Npc1^-/-^ retina.

## Conclusions

In the Npc1^-/-^ retina, accumulation of electron-dense inclusions, ectopic processes and defect arborisation were found in different types of cells accompanied by hyperactivity of glial cells, suggesting multiple cellular defects in the retina of the Npc1 mutant mouse.

## Additional file

Additional file 1: Figure S1. Activated glial cells in the Npc1^-/-^ retina demonstrated by immunostaining with antibodies against GFAP for astrocytes and F4/80 for microglial cells at different stages (P2, P9 and P30). Abbreviations: ONL, outer nuclear layer; OPL, outer plexiform layer; INL, inner nuclear layer; IPL, inner plexiform layer; GCL, ganglion cell layer. Scale bar: 200 μm in A for all.

## Abbreviations

CNS: Central nervous system
Goα: Alpha subunit of G protein
GCL: Ganglion cell layer
GFAP: Glial fibrillary acidic protein
GS: Glutamine synthetase
INL: Inner nuclear layer
IPL: Inner plexiform layer
NeuN: Hexaribonucleotide binding protein-3
NPC1: Niemann-pick type C1
ONL: Outer nuclear layer
OPL: Outer plexiform layer
P: Postnatal day
PBS: Phosphate-buffered saline
PFA: Paraformaldehyde
PKCα: Alpha isoform of protein kinase C
RGC: Retinal ganglion cell
TH: Tyrosine hydroxylase
TUNEL: Terminal deoxynucleotidyl transferase dUTP nick end labelling
