# Supplementary figures and images for: Defects in the retina of Niemann-pick type C 1 mutant mice
Source: BMC Neurosci. 2014 Nov 29;15:126. doi: 10.1186/s12868-014-0126-2 (PMC4267119; doi:10.1186/s12868-014-0126-2)

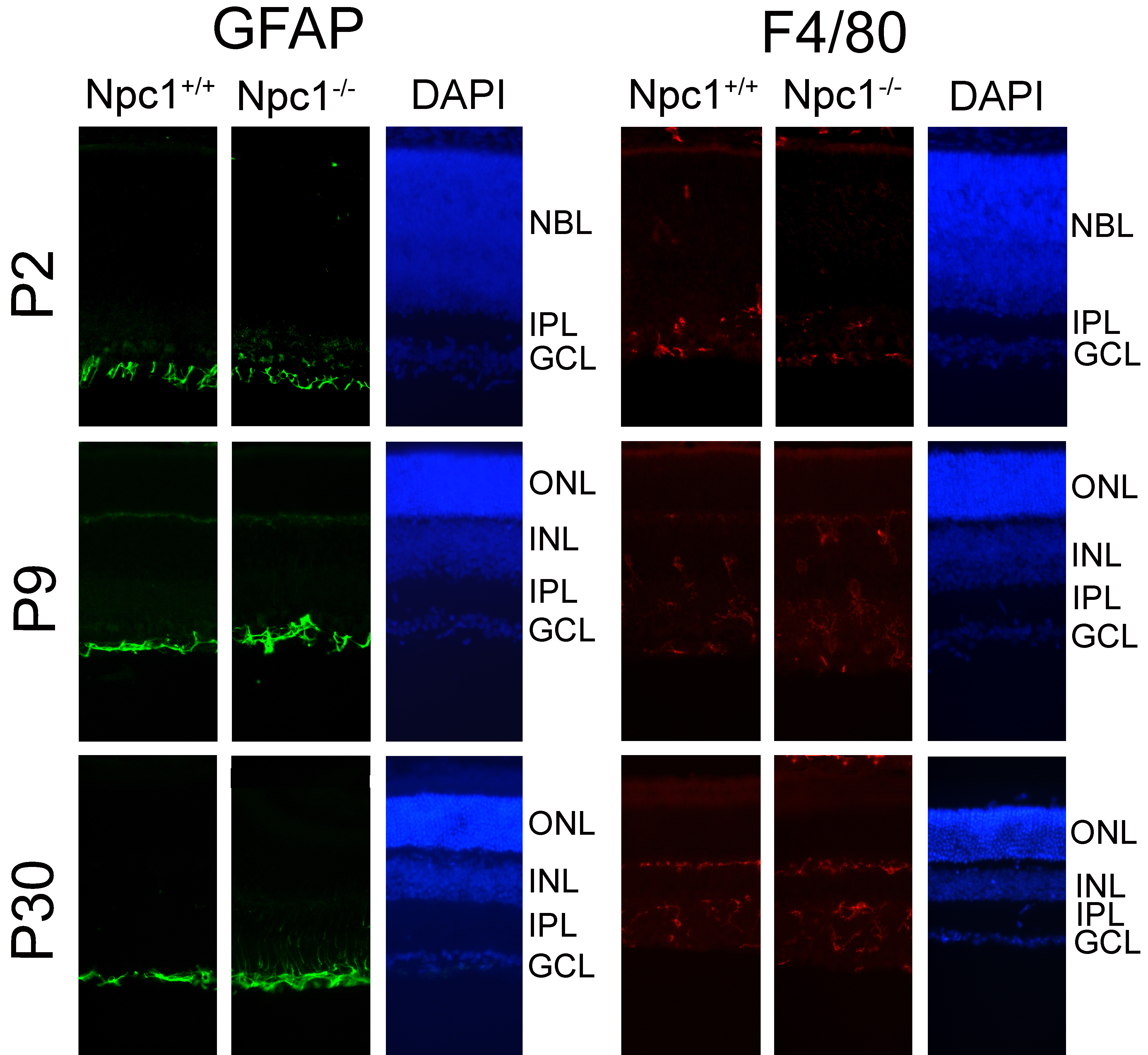

Supplement: Additional file 1: Figure S1. — Activated glial cells in the Npc1-/- retina demonstrated by immunostaining with antibodies against GFAP for astrocytes and F4/80 for microglial cells at different stages (P2, P9 and P30). Abbreviations: ONL, outer nuclear layer; OPL, outer plexiform layer; INL, inner nuclear layer; IPL, inner plexiform layer; GCL, ganglion cell layer. Scale bar: 200 μm in A for all. [file 12868_2014_126_MOESM1_ESM.jpeg]
